# Supplementary material for: Low melt viscosity enables melt doublets above the 410-km discontinuity
Source: Nat Commun. 2025 Apr 4;16:3239. doi: 10.1038/s41467-025-58518-7 (PMC11971259; doi:10.1038/s41467-025-58518-7)
Supplement: Supplementary file 1 — Supplementary Information [file 41467_2025_58518_MOESM1_ESM.pdf]

Supplementary Materials for "Low Melt Viscosity Enables Melt Doublets Above The 410-km Discontinuity"

Longjian Xie[1,2*], Denis Andrault[3], Takashi Yoshino[4], Cunrui Han[5], James O. S. Hammond[5], Fang Xu[6], Bin Zhao[4], Oliver T. Lord[7], Yingwei Fei[8], Simon Falvard[3], Sho Kakizawa[9], Noriyoshi Tsujino[9], Yuji Higo[9], Laura Henry[10], Nicolas Guignot[10], David P Dobson[2*]

1. Center for High Pressure Science & Technology Advanced Research, Shanghai, 201203, China
2. *Department of Earth Sciences, University College London, London WC1E6BS, UK.*
3. *Université Clermont Auvergne, CNRS, IRD, OPGC, Laboratoire Magmas et Volcans, F‑63000, Clermont‑Ferrand, France*
4. *Institute for Planetary Materials, Okayama University, Misasa, Tottori 682-0193, Japan*
5. *School of Natural Sciences, Birkbeck, University of London, Malet Street, WC1E 7HX, UK*
6. *School of Earth Sciences, Zhejiang University, Hangzhou 310058, China*
7. *School of Earth Sciences, University of Bristol, Bristol, BS81RJ, UK*
8. *Earth & Planets Laboratory, Carnegie Institution for Science, Washington DC 20015, USA.*
9. *Japan Synchrotron Radiation Research Institute, 1-1-1 Kouto, Sayo, Hyogo 689-5198, Japan.*
10. *Synchrotron SOLEIL, Gif-sur-Yvette, France.*

*Corresponding author.
E-mail: ddtuteng@gmail.com

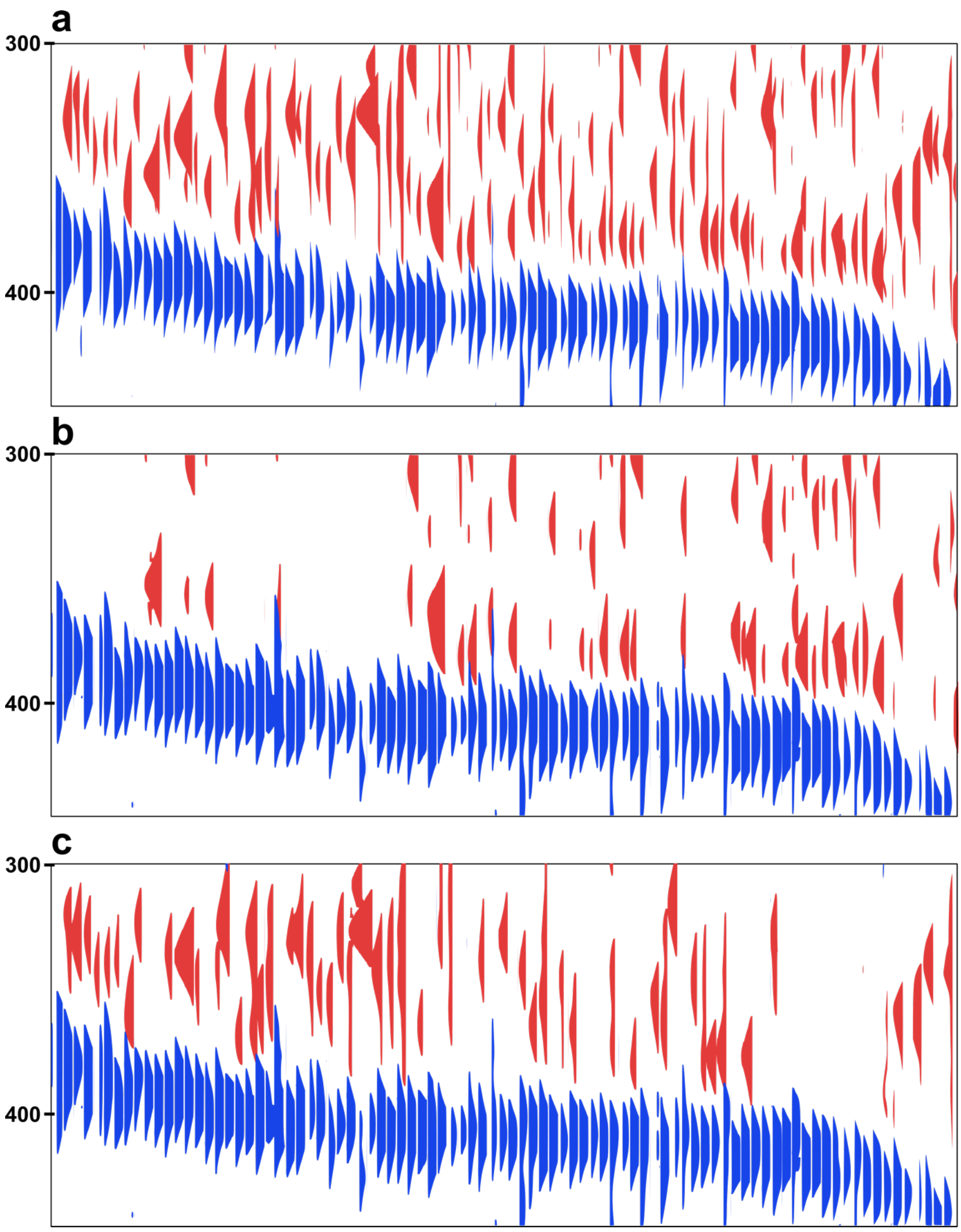

Supplementary Fig. 1. Single LVLs and LVL doublets observed under individual stations. **a.** 89 stations show a negative signal atop 410, according to Tauzin et al. (2010) [1]. **b.** Stations from **a** showing two negative signals atop 410. **c.** Stations from **a** showing one negative signal atop 410. Red and blue colors indicate negative and positive phases, respectively. To highlight the negative signals, the positive phases above 410 are removed.

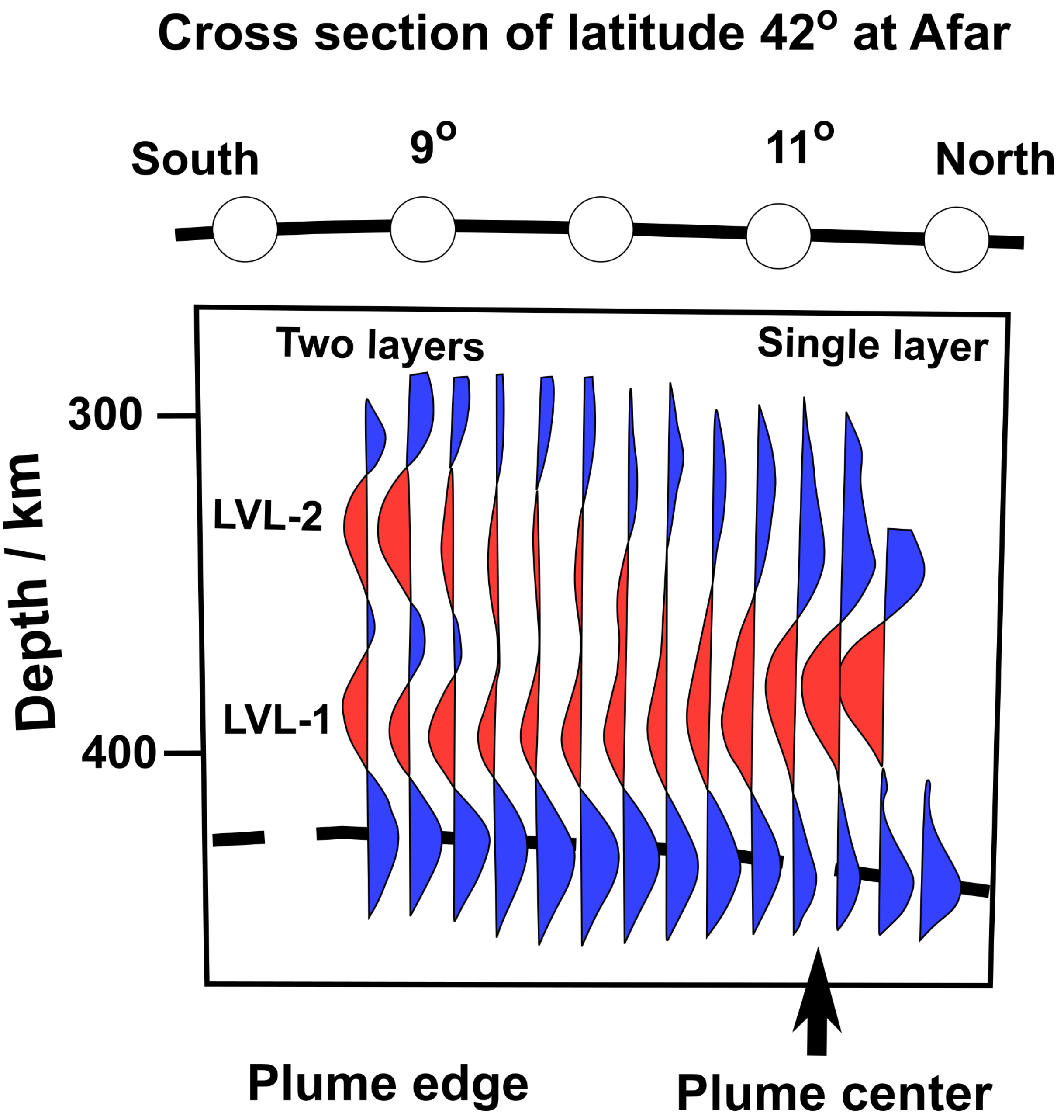

Supplementary Fig. 2. Conversion between single LVL and LVL doublets underneath Afar Triple Junction, modified from Thompson et al. (2015) [2]. Red and blue colors indicate negative and positive phases, respectively.

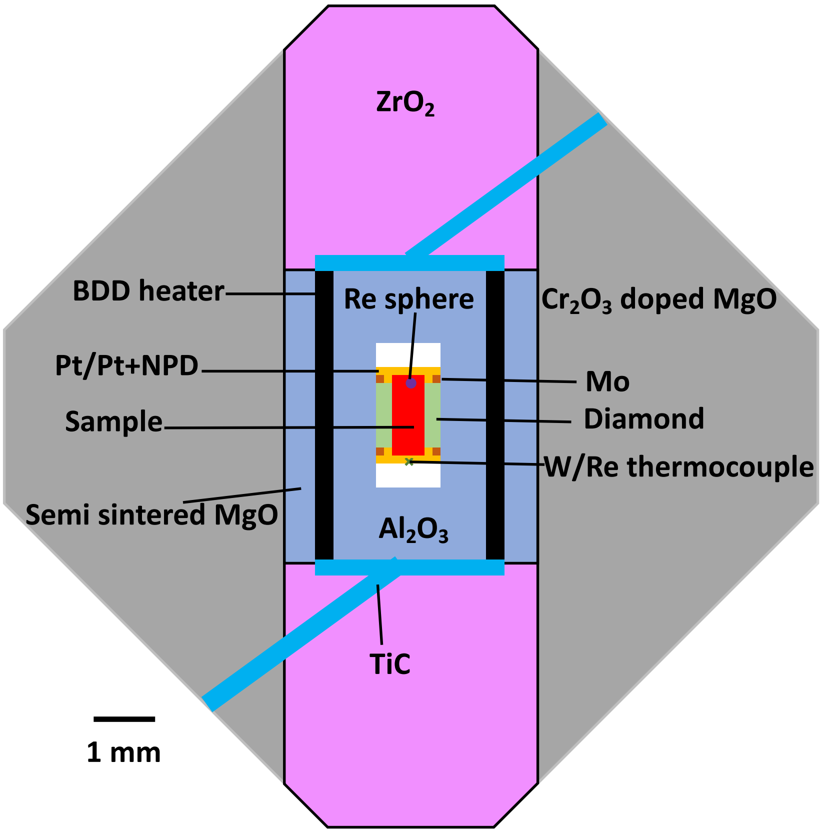

Supplementary Fig. 3. Cross-sectional view of the cell assembly aligned parallel to the X-ray beam for viscosity measurements.

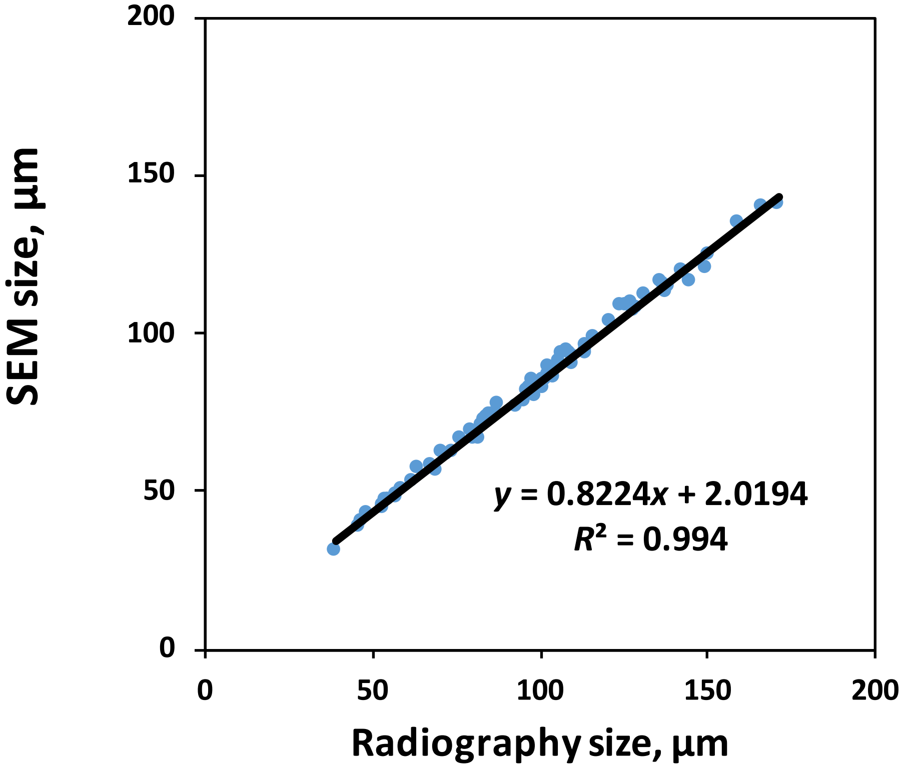

Supplementary Fig. 4. Correlation between the diameters of Re spheres measured by radiographic and SEM methods. Source data are provided as a Source Data file.

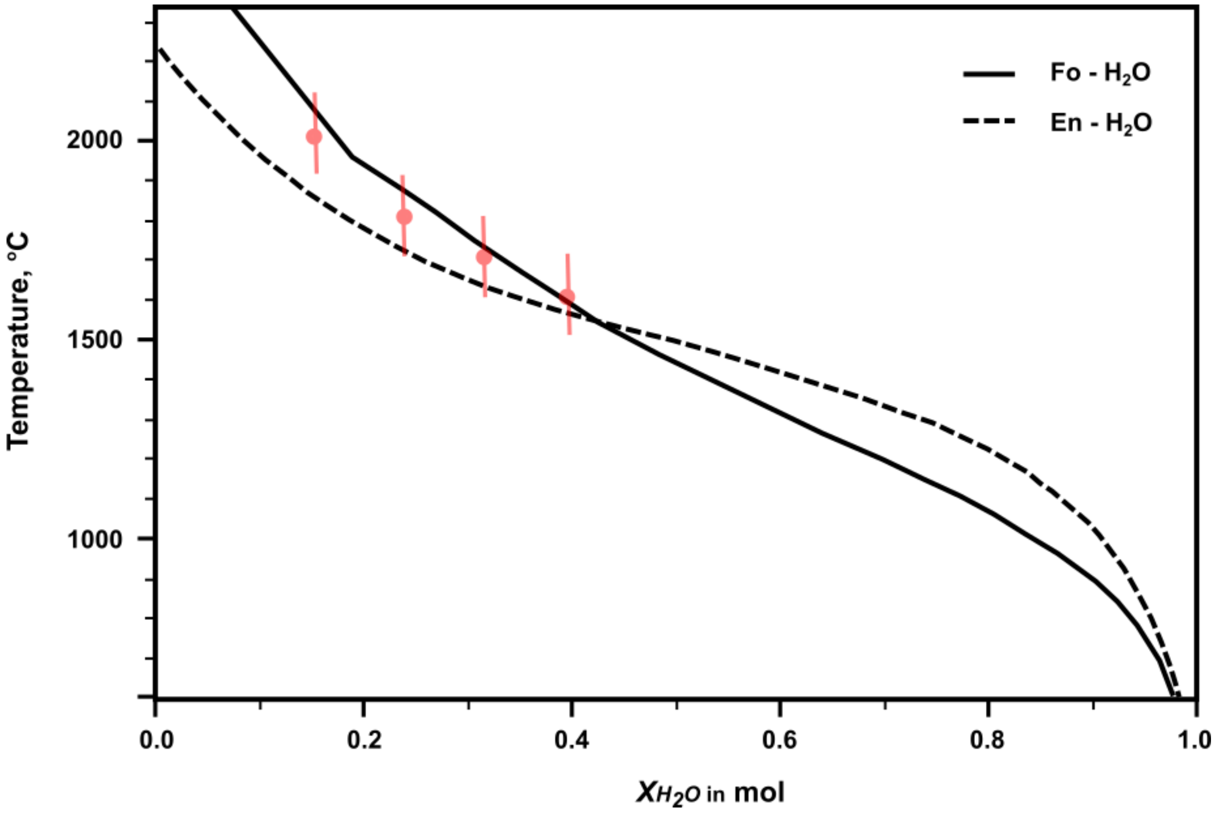

Supplementary Fig. 5. Temperatures during sphere falls. Red circle indicates the temperatures from this study. The vertical red segments represent the error bars in 1 sigma. For comparison, the liquidus of forsterite (Fo) -H_2O and enstatite (En) - H_2O systems[3] are plotted as solid and dashed lines, respectively. Source data are provided as a Source Data file.

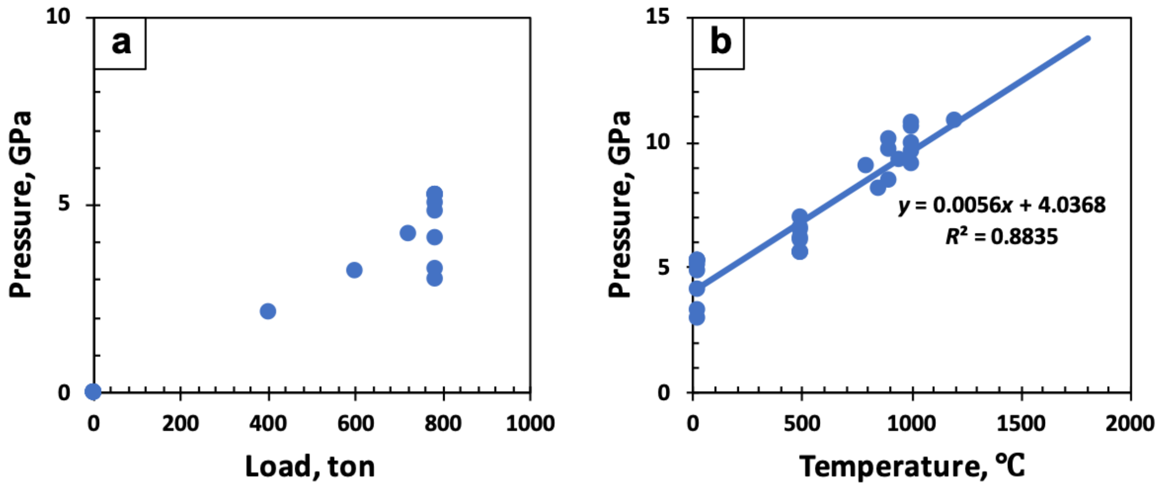

Supplementary Fig. 6. (a) Sample pressure during compression at 300 K and (b) sample pressure including thermal pressure during heating at 780 tons (b). Source data are provided as a Source Data file.

repl

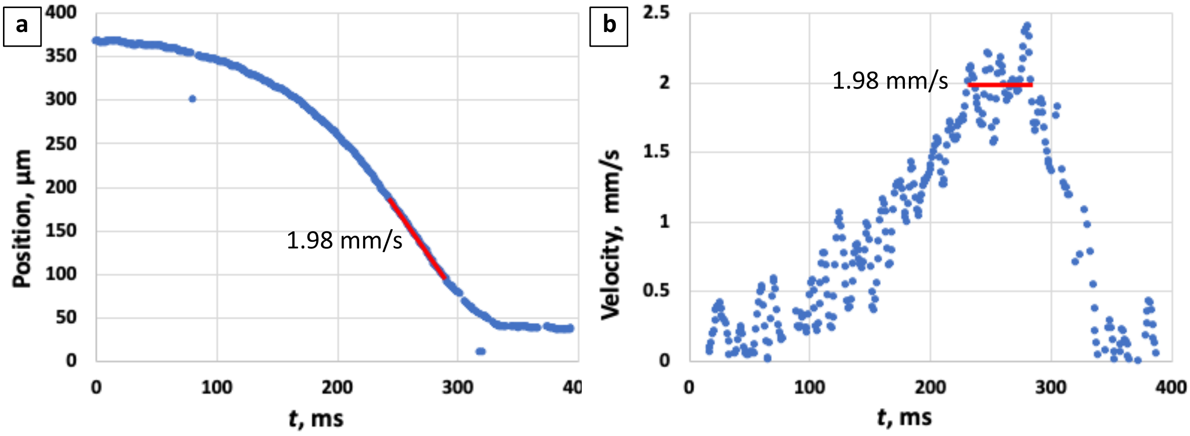

Supplementary Fig. 7. The position-time (a) and velocity-time (b) diagram of Run S3641. The red line marks the region where the sphere reached the terminal velocity. Source data are provided as a Source Data file.

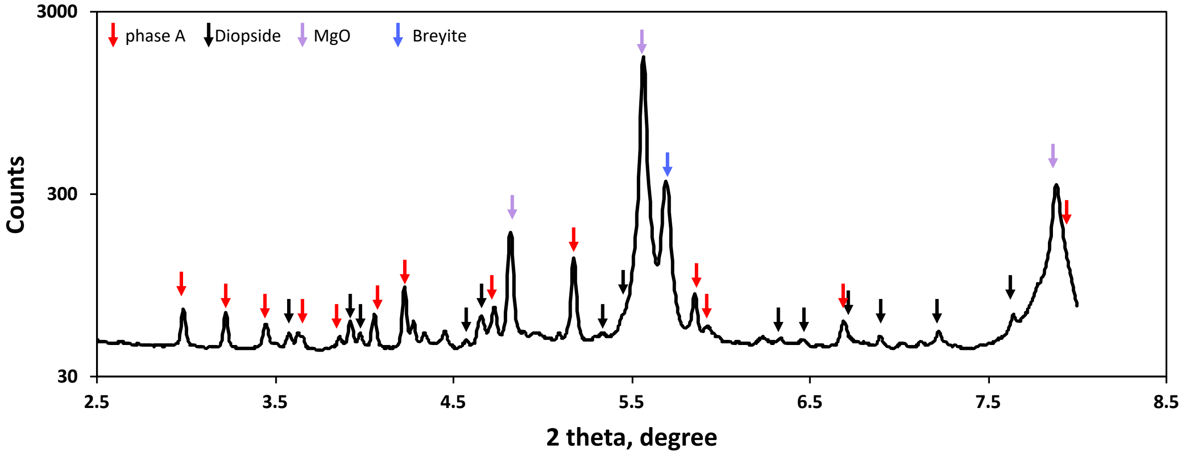

Supplementary Fig. 8. The X-ray diffraction pattern of the recovered sample from Run S3642.

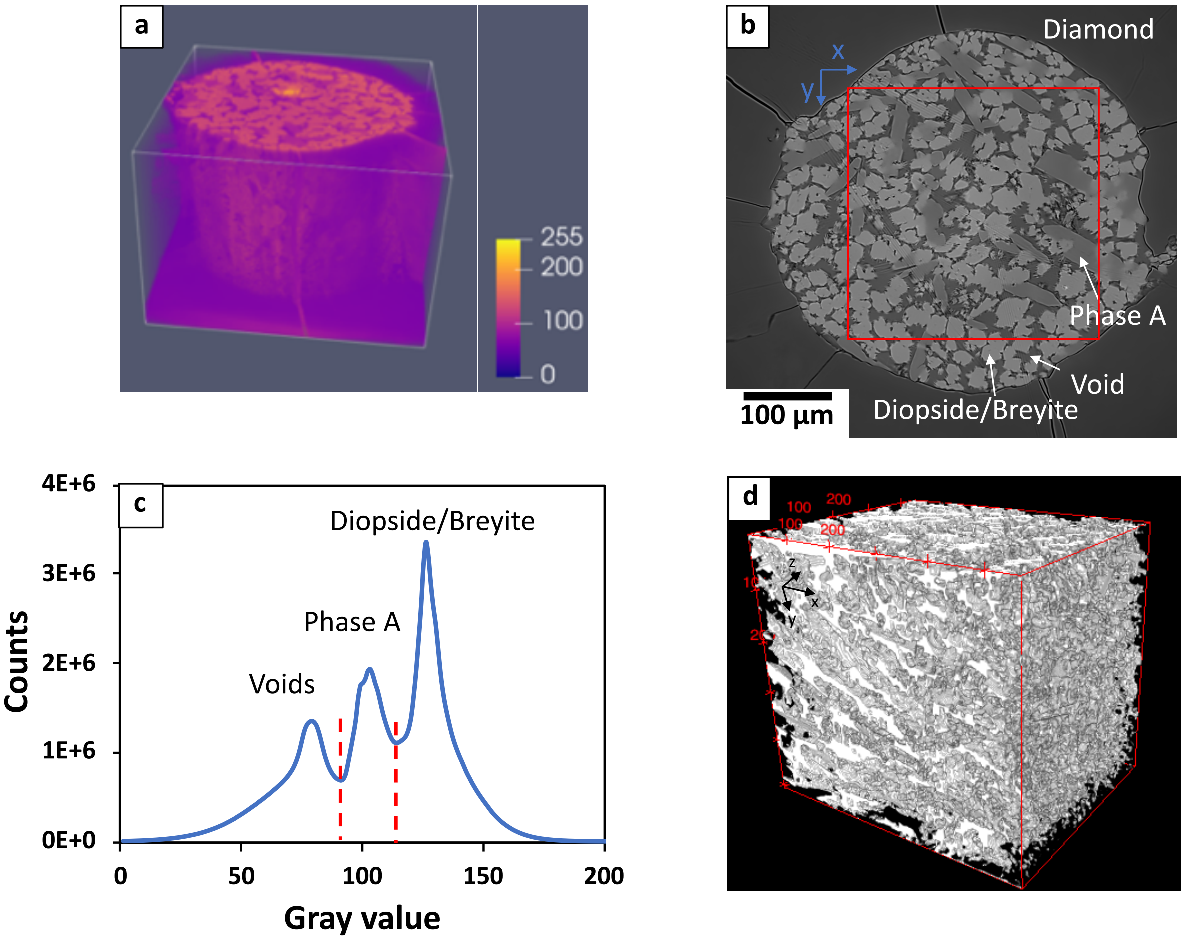

Supplementary Fig. 9. Example tomography results of the recovered sample from Run S3641. a. Reconstructed 3D volume of sample. **b.** A slice of the X-ray absorption coefficient contrast image. **c.** Histogram plot of gray values within the rectangular region shown in panel **b**. The dashed red lines mark the thresholds for isolating phases. **d.** 3D distribution of voids within the rectangular area shown in panel **b**.

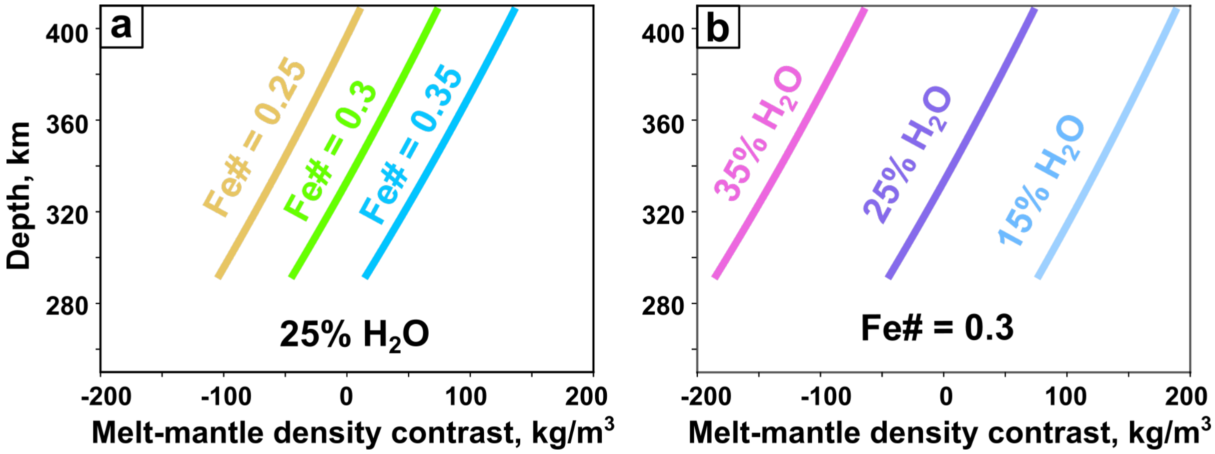

Supplementary Fig. 10. Relation between depth and melt-mantle density contrast. a. Relation against variable Fe# at fixed 25% H_2O. **b.** Relation against variable water contents at fixed Fe# = 0.3. The melt density is calculated according to the equation of state of hydrous silicate melt[4].

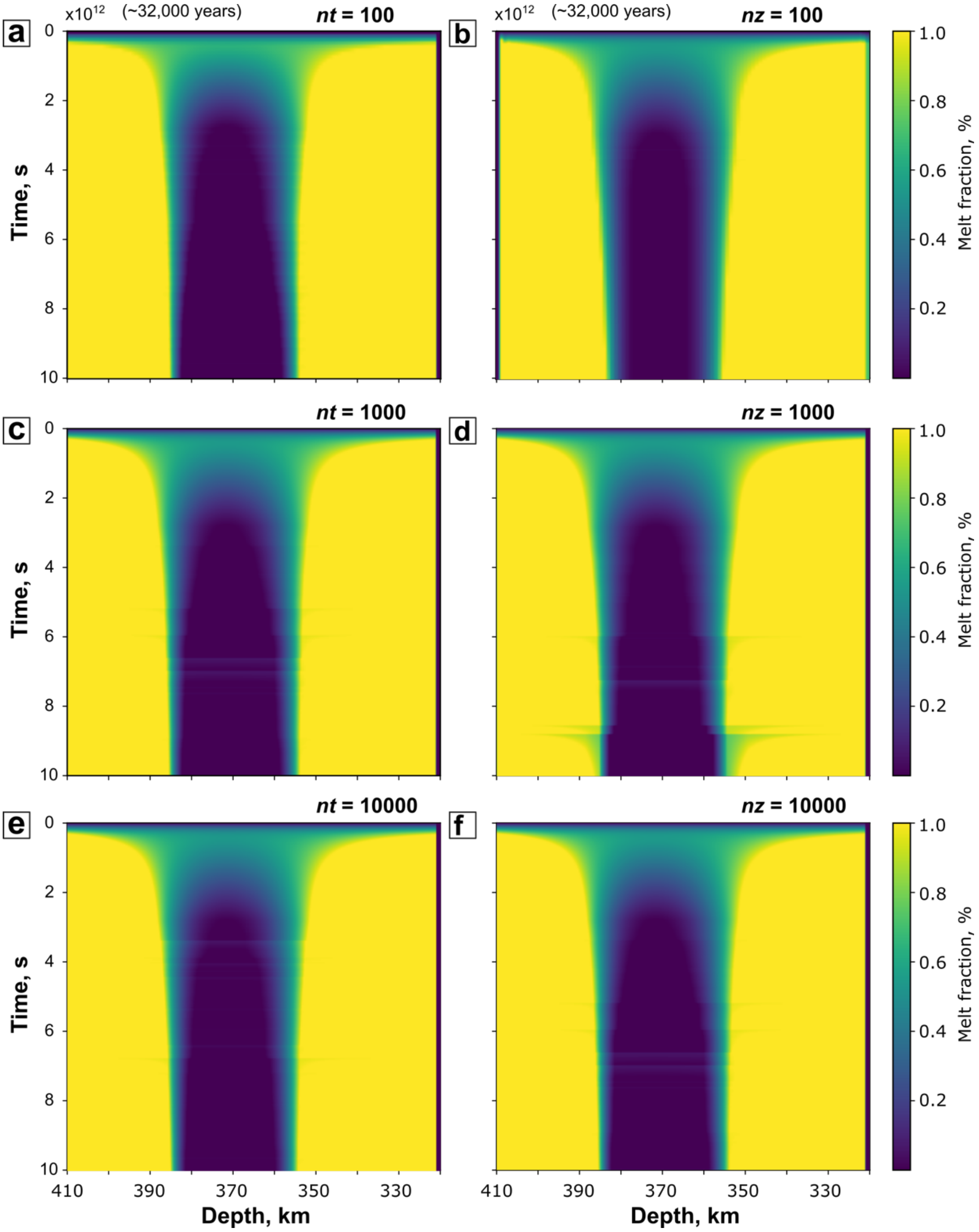

Supplementary Fig. 11. Effect of mesh size in space and time on the simulation results. The left panels (**a**, **c**, **e**) display results calculated using a mesh number of 10000 for the spatial domain, with mesh number of 100, 1000, 10000 for the time steps (*nt*), respectively. The right panels (**b**, **d**, **f**) show results calculated using a mesh number of 1000 for the time steps, with mesh number of 1000, 10000, 50000 for the spatial domain (*nz*), respectively. The other parameters are provided in Suppl. Table 4.

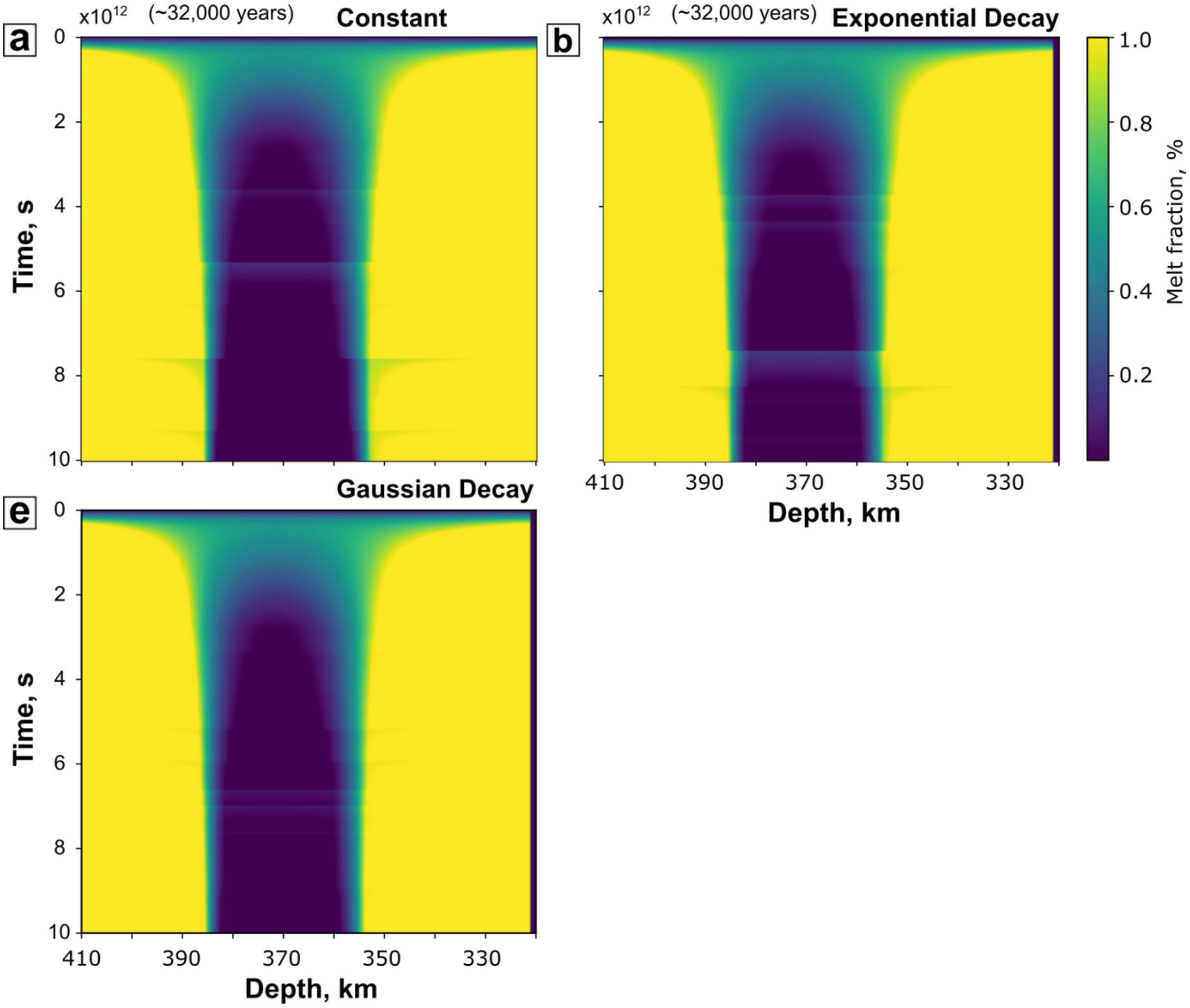

Supplementary Fig. 12. Effect of boundary conditions on the simulation results. Panels **(a**, **b**, **c)** show results using Constant, Exponential decay and Gaussian decay boundary conditions, respectively. All other controlling parameters are as reported in Suppl. Table 4. Comparison between the graphs show little impact on the calculations of the boundary conditions between Constant, Exponential decay and Gaussian decay.

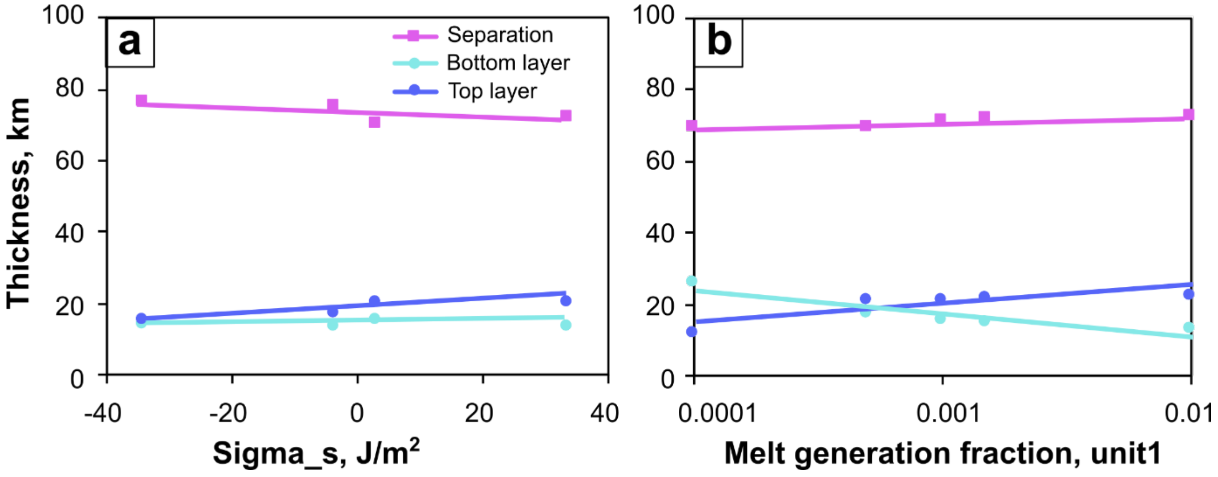

Supplementary Fig. 13. Minor factors on the thickness of the melt layers and thickness of the regions in-between them (separation) in a CDM model. a. Effect of surface tension. Sigma_s: defined as $-3.3908\sigma\_mf+2.1587\sigma\_mm$ is the term of bulk surface tension in Eq. (11). **b.** Effect of fraction of melt generated upon mantle unwilling. The values of all other controlling parameters can be found in Suppl. Table 4. Source data are provided as a Source Data file.

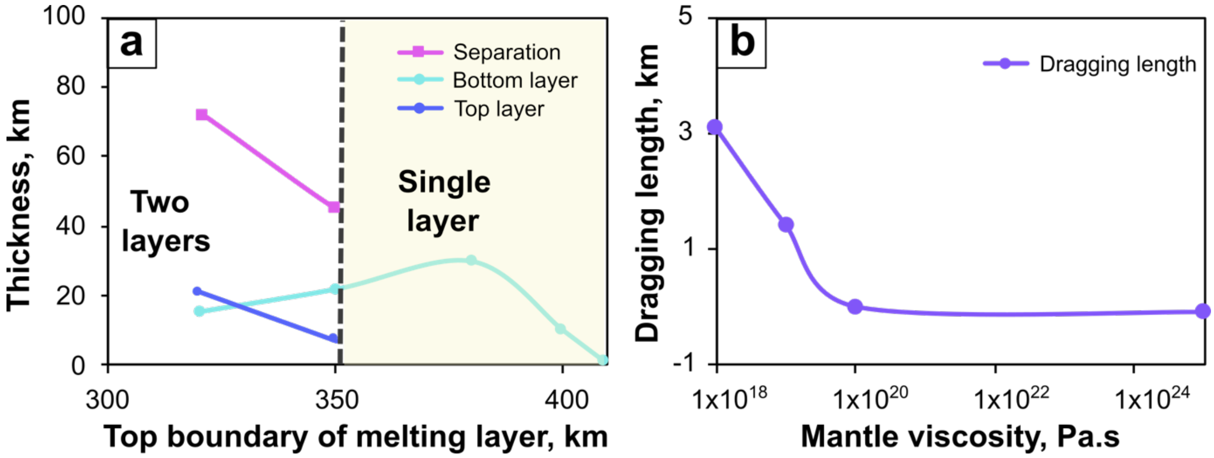

Supplementary Fig. 14. a. The boundary between a AT and CDM model in terms of the thickness of melt generating layer. b. The effect of mantle viscosity on the dragging length and the layer pattern. The dragging length is calculated by subtracting the melt generating layer thickness from the stabilized layer thickness. The thickness of the melt generating layer is set to be 1 km. The values of all other controlling parameters can be found in Suppl. Table 4. Only 1 bottom layer exists suggesting that the AT model does not change to a CDM model within the typical range of mantle viscosity. Source data are provided as a Source Data file.

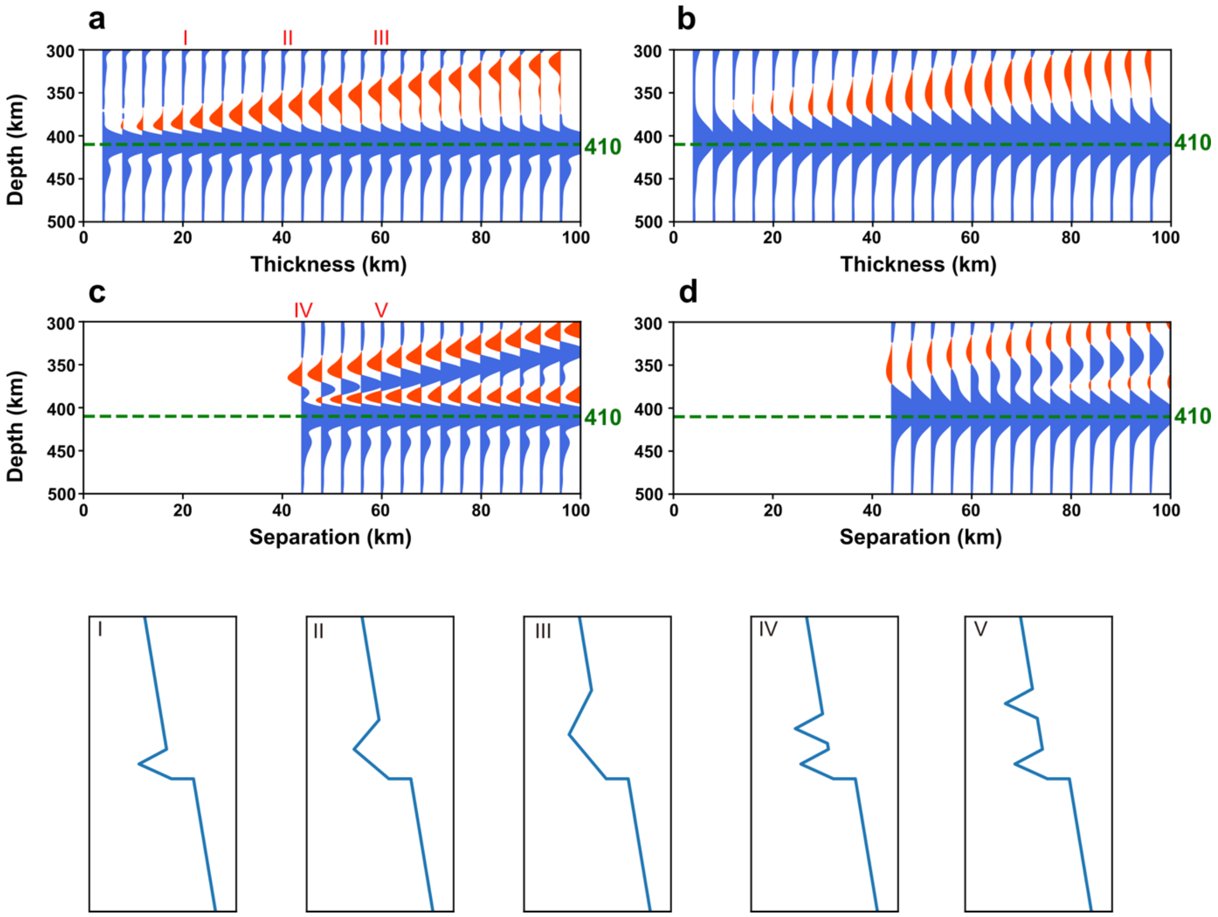

Supplementary Fig. 15. Synthetic receiver function models for single (models I, II and III) and double melt layers (models IV and V). a. synthetic receiver functions showing a single layer with Gaussian pulse width of 2 s. **b.** synthetic receiver functions showing a single layer with Gaussian width of 4 s. In **a.** and **b.**, thickness refers to the thickness of the layer. **c.** synthetic receiver functions showing a double melt layer with Gaussian pulse width of 2 s. **d.** synthetic receiver functions showing a double melt layer with Gaussian pulse width of 4 s. In **c.** and **d.**, separation refers to the distance between the top boundaries of the two melt layers.

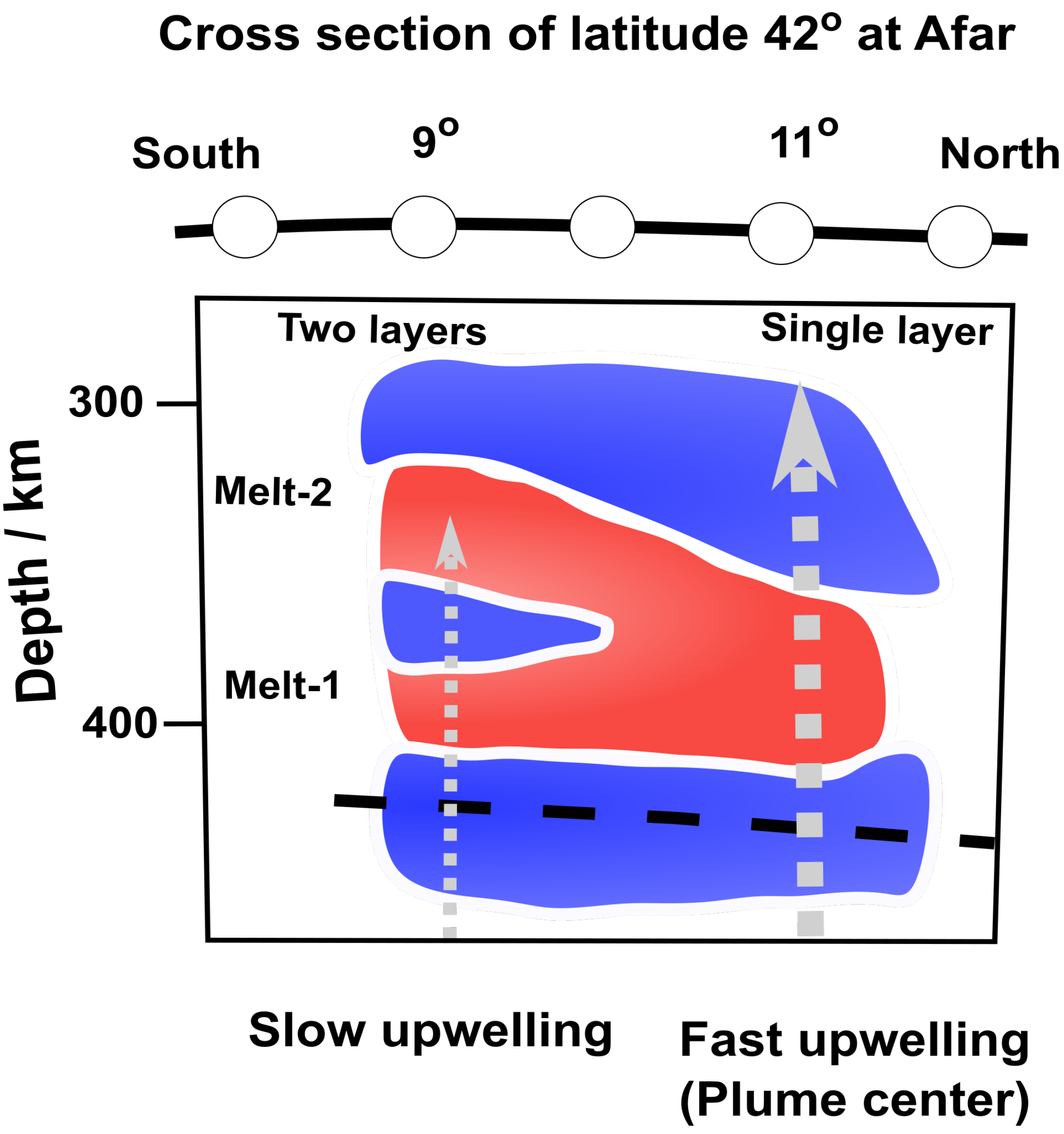

Supplementary Fig. 16. Relationship between melt layer pattern and upwelling rate at Afar Triple Junction. The figure was modified from Thompson et al. (2015) [2].

Supplementary Table 1. The composition of starting materials in wt%.

Oxides	Sample with 15.5 mol% H_2O	Sample with 24.2 mol% H_2O	Sample with 31.8 mol% H_2O
**SiO_2**	35.14	33.87	32.02
**Al_2O_3**	0.79	0.77	0.72
**MgO**	40.66	38.32	37.05
**CaO**	17.13	16.51	15.61
**H_2O**	6.28	10.54	14.60
**total**	100.00	100.01	100.00

Supplementary Table 2. Summary of experimental conditions and results

Run #	H_2O (wt%)	H_2O (mol%)	P (GPa)	T (°C)	ds^+ (µm)	err of ds	v_s (mm s^{-1})	η (mPa·s)	err of η
S3641	14.60	31.8	13.6	1700	55.5	1.8	0.00198	12.1	0.6
S3642	10.54	24.2	14	1800	61.4	4.5	0.00108	26.2	2.8
S3645	6.28	15.5	15	2000	103.3	4	0.000628	104.0	5

+ Diameter of probe spheres

Supplementary Table 3. Volumes of phases and water content estimated using reconstructed 3D tomography models

Run #	Initial H_2O (wt%)	Void (vol.%)	Diopside (vol.%)	Phase A (vol.%)	H_2O in recovered sample (wt%)
S3641	14.60	21.9	41.8	36.3	13
S3642	10.54	--	--	--	--
S3645	6.28	1.3	46.4	52.3	7

Supplementary Table 4. Parameters used for 1D FEM simulation.

Total time, s	1×10^{12} - 1×10^{13} (1×10^{13}) *
Total time steps	1000
Mesh number	10000
Depth of NDP, km	320 – 400 (320) *
Mantle upwelling rate, mm/year	1 – 10 (1) *
Viscosity of melt, Pa·s	0.02
$\Delta\rho$ at 410 km, kg/m^3	0 – 300 (150) *
Mantle viscosity, Pa·s	$1 \text{ x } 10^{18}$ - $1 \text{ x } 10^{21}$ (1×10^{20}) *
Initial melt fraction	$1 \text{ x } 10^{-6}$
g, m/s^2	9.8
d, m	0.001
K0	4/3
f	0.001
σ_s =(-3.3908σ_mf+2.1587σ_mm), J/m^2	-10 – 10 (3.4) *

* Unless stated otherwise, the parameters in the brackets are used for all the calculations.

References

1. Tauzin, B., Debayle, E. & Wittlinger, G. Seismic evidence for a global low-velocity layer within the Earth's upper mantle. *Nat Geosci* **3**, 718–721 (2010).
2. Thompson, D. A. *et al.* Hydrous upwelling across the mantle transition zone beneath the Afar Triple Junction. *Geochemistry, Geophysics, Geosystems* **16**, 834–846 (2015).
3. Myhill, R., Frost, D. J. & Novella, D. Hydrous melting and partitioning in and above the mantle transition zone: Insights from water-rich $MgO–SiO_2–H_2O$ experiments. *Geochim Cosmochim Acta* **200**, 408–421 (2017).
4. Drewitt, J. W. E., Walter, M. J., Brodholt, J. P., Muir, J. M. R. & Lord, O. T. Hydrous silicate melts and the deep mantle H_2O cycle. *Earth Planet Sci Lett* **581**, (2022).
